# Metabolic mechanisms of acute proximal tubular injury

**DOI:** 10.1007/s00424-022-02701-y

**Published:** 2022-05-14

**Authors:** Andrew M. Hall, Sophie de Seigneux

**Affiliations:** 1 Institute of Anatomy, University of Zurich, Winterthurerstrasse 190, 8057 Zurich, Switzerland; 2 Department of Nephrology, University Hospital Zurich, Zurich, Switzerland; 3 Department of Cell Physiology and Metabolism, University of Geneva, Geneva, Switzerland; 4 Department of Medicine, Service of Nephrology, Geneva University Hospitals, Geneva, Switzerland

**Keywords:** Proximal tubule, Metabolism, Mitochondria, Acute kidney injury

## Abstract

Damage to the proximal tubule (PT) is the most frequent cause of acute kidney injury (AKI) in humans. Diagnostic and treatment options for AKI are currently limited, and a deeper understanding of pathogenic mechanisms at a cellular level is required to rectify this situation. Metabolism in the PT is complex and closely coupled to solute transport function. Recent studies have shown that major changes in PT metabolism occur during AKI and have highlighted some potential targets for intervention. However, translating these insights into effective new therapies still represents a substantial challenge. In this article, in addition to providing a brief overview of the current state of the field, we will highlight three emerging areas that we feel are worthy of greater attention. First, we will discuss the role of axial heterogeneity in cellular function along the PT in determining baseline susceptibility to different metabolic hits. Second, we will emphasize that elucidating insult specific pathogenic mechanisms will likely be critical in devising more personalized treatments for AKI. Finally, we will argue that uncovering links between tubular metabolism and whole-body homeostasis will identify new strategies to try to reduce the considerable morbidity and mortality associated with AKI. These concepts will be illustrated by examples of recent studies emanating from the authors’ laboratories and performed under the auspices of the Swiss National Competence Center for Kidney Research (NCCR Kidney.ch).

## Introduction

Acute kidney injury (AKI) is defined as an abrupt loss of kidney function, manifesting clinically as a rapid increase in blood markers of filtration (e.g., creatinine) and/or a decrease in urine output. AKI occurs in 10–15% of patients admitted to hospital and is associated with substantial morbidity, mortality, and financial burdens on health systems [101]. Aside from specific other causes like glomerulonephritis and post-renal obstruction, most cases of AKI result from insults to the proximal tubule (PT), including ischemia/hypoxia, sepsis, and toxicity from xenobiotics (e.g., drugs or heavy metals) or endogenous proteins (e.g., myoglobin in rhabdomyolysis, immunoglobin light chains in plasma dyscrasias).

The PT is the workhorse of the kidney, performing the bulk of fluid reabsorption post glomerular filtration. Metabolism within PT cells is closely coupled to solute transport function, and substantial changes in metabolism occur during AKI. PTs are densely packed with elongated and interconnected mitochondria, which are needed to generate ATP to drive the movement of solutes, and most of the aforementioned insults target these organelles. It therefore follows that deepening understanding of the metabolic/mitochondrial changes that occur could open the way to developing new therapeutic strategies. Accordingly, there has been an explosion of research on this topic in the last few years, and a number of recent in-depth review articles have nicely covered this [5, 17, 25, 49, 62, 64, 65, 107]. Our intention here is not to simply repeat the valuable information contained within these *verbatim*, but rather to introduce the subject and succinctly summarize the current state of the field. We will then proceed to highlight three broad concepts that have received rather less attention to date, but might nevertheless be important for translating basic AKI research into the clinic.

## Basic physiology of the proximal tubule

### Solute transport

The PT begins immediately after the glomerulus and reabsorbs numerous filtered solutes to prevent their loss in the urine. Vectorial transport is driven by the highly abundant basolateral Na^+^/K^+^-ATPase transporter [15], which is the major consumer of ATP in the PT. The movement of various solutes — including glucose, amino acids, phosphate, and bicarbonate — is then either directly or indirectly coupled to that of sodium and involves an array of membrane co-transporters. In addition, a substantial amount of fluid is also reabsorbed via the leaky paracellular route. Another specialized function of the PT is to reclaim small filtered plasma proteins and their cargo, via receptor mediated endocytosis [85]. In parallel to its resorptive function, the PT also secretes organic solutes that are poorly filtered (usually due to albumin binding), using various organic anion and cation transporters (OATs and OCTs, respectively) [123]. These have long been studied in the context of renal drug handling, but interest is also growing in their role in excreting putative uremic toxins, some of which are generated by the gut microbiome [117].

The PT is divided anatomically into convoluted and straight parts, the latter entering the medulla. It can also be classified as having three distinct segments (termed S1-3), based on subtle differences in cellular ultrastructure visible with electron microscopy [15]. This segmentation is observed in various different species, including rodents, rabbits, dogs, and primates [15]. Previous micro-puncture studies in rats have suggested — somewhat intuitively — that reabsorption of filtered solutes is greatest in the early part of the PT [71]. Meanwhile, experiments performed in isolated segments of rabbit PT revealed a higher secretion of organic anions in more distal regions [122]. These findings suggest that solute transport is somehow spatially organized along the PT, and that changes in cell morphology probably reflect adaptation to sub-specialized tasks. But the extrinsic factors that shape these axial patterns, and to what extent specific transport mechanisms map exclusively to individual segments, remains far from clear [38] (although answers are starting to emerge from gene expression and functional imaging studies — see below). Moreover, it needs to be determined whether structure-function relationships in the PT of laboratory animals are fully replicated in humans.

### Metabolism

The high transport demands placed on the PT necessitate a rich and constant supply of ATP, which is provided by mitochondria. Accordingly, the kidney is second only to the heart in terms of mitochondrial density [5]. It has long been known that renal tubular oxygen consumption displays a linear relationship with sodium transport [72], suggesting very tight coupling between the two. Previous studies have suggested that the PT is almost entirely dependent on aerobic metabolism to produce ATP and that — unlike distal tubular segments — it lacks substantial glycolytic capacity [4]. While aerobic respiration is undoubtedly more efficient, the lack of a backup option renders the PT extremely vulnerable to mitochondrial insults; this concept is exemplified by the high prevalence of PT transport defects in patients with genetic disorders in mitochondrial function [74].

Older studies performed with isolated nephron segments demonstrated that PTs can metabolize an impressively diverse range of substrates, including fatty acids (FAs), ketone bodies, amino acids, pyruvate, lactate, and citric acid cycle intermediates [121]. It is often stated that FAs are the preferred fuel for PTs [30, 51], and it is certainly true that — pound for pound — they produce the highest yield of ATP. However, this concept overlooks the importance of substrate availability within a complex filtering organ and the increasing experimental evidence of metabolic heterogeneity along the PT (see later).

In addition to ATP generation, there are a number of other specialized metabolic processes that also take place within the PT, including plasma protein catabolism, arginine metabolism, ammoniagenesis, the urea cycle, and detoxification/conjugation of xenobiotics and organic solutes. Once again, we unfortunately lack a detailed understanding of the spatial arrangement of these processes along the PT, and how they might be integrated with other functions. Together with the liver, the PT is also a major site of glucose production (gluconeogenesis), which provides an explanation as to why reabsorbed glucose is apparently not used by the PT as a metabolic fuel (i.e., to avoid a futile cycle). During AKI, the loss of tubular metabolic functions with direct relevance to whole-body homeostasis, such as gluconeogenesis, might help to explain high levels of associated morbidity and mortality.

## Metabolic and mitochondrial responses to acute proximal tubular injury

Much has been learnt in recent years concerning metabolic and mitochondrial changes that occur in PT cells in response to acute injury, which has uncovered a raft of potential new targets for therapeutic intervention. Most of this information has been derived from ischemia-reperfusion models in rodents, and to a lesser extent cisplatin toxicity. Since this topic has been covered in detail by others, we will simply provide a summary of major findings here.

Acute cessation of aerobic respiration in the PT typically causes extensive fragmentation of the mitochondrial network [37, 111] and a rapid decline in intracellular ATP [125]. The GTPase dynamin-1-like protein (Drp1) probably plays an important role in mitochondrial fission during AKI, and blocking it seems to be protective [10, 67, 94]. Mitochondrial injury often leads to oxidative stress, especially during the post-ischemic reperfusion phase when the oxygen supply is restored. When severe, this can trigger mitochondrial swelling, perhaps in part due to opening of a large non-specific pore in the inner membrane (the mitochondrial permeability transition pore — mPTP) [110]. Mitochondrial targeted anti-oxidants have been developed, which have shown promise in pre-clinical AKI studies [2, 53, 109], and are now entering human trials [64]. However, some caution may be required, since the chemical properties that enable these agents to target mitochondria can also lead to some unwanted side effects [32].

ATP depletion induces acute changes in PT cell morphology, including retraction of the brush border, membrane blebbing, and shedding of cellular debris into the lumen [31], resulting in the appearance of a flattened epithelia, the histological hallmark of AKI. Loss of normal calcium homeostasis results in large rises in intracellular calcium concentration [75, 119], which might in part drive some of these structural alterations, including rearrangement of the actin cytoskeleton. Major changes in intracellular lipid content also occur [27], likely driven at least in part by a decrease in consumption as a metabolic fuel. Moreover, the energetic crisis of AKI triggers organelle degradation by autophagy, and enhancing the specific removal of damaged mitochondria (mitophagy) might help to limit oxidative stress [118].

Post injury, flattened PT cells dedifferentiate and entering a quasi-dormant state, presumably reflecting a survival strategy to reduce ATP demands, and prevent uncontrolled and immunogenic cell death. There is some evidence that during this effective hibernation, they increase glycolytic capacity [58], before eventually re-differentiating during the recovery phase of AKI, the transcriptional regulation of which is currently the subject of intense research [56]. Large numbers of epithelial cells are also shed into the urine, leading to a substantial decrease in the remaining viable population [60]. Although mitochondrial damage can trigger apoptosis, this does not seem to be a predominant feature in most types of AKI [73]. However, other types of cell death, such as ferroptosis and necroptosis, probably contribute more substantially to tubular injury [73]. Of note, ferroptosis is triggered by oxidative stress (alterations in glutathione concentration/redox state and lipid peroxidation), which, as already stated, can arise from mitochondrial dysfunction.

Failure of surviving PT cells to re-differentiate triggers the release of pro-fibrotic factors and probably represents a critical watershed between reversible damage and the development of progressive chronic kidney disease (CKD) [42]. Several studies have shown that mitochondrial biogenesis is temporarily downregulated in the PT in response to injury, at least in part due to a decreased expression of the master regulator peroxisome proliferator-activated receptor gamma coactivator 1-alpha (PGC1α) [62, 69]. There has therefore been much interest in developing compounds that might boost PGC1α activity at the appropriate time point to hasten recovery [5]. Meanwhile, the release of mtDNA from damaged organelles might be important in triggering secondary inflammatory responses [16].

Finally, a landmark study in the field identified that abrupt decreases in intracellular nicotinamide adenine dinucleotide (NAD) occur during AKI, also probably linked to suppression of mitochondrial biogenesis [114]. NAD is a redox co-factor involved in many different critical metabolic processes, including beta oxidation, the citric acid cycle, and the respiratory chain. Therefore, restoring NAD function could have myriad beneficial effects and might be substantially more potent than targeting a single pathway [52, 97]. Moreover, supplementation with precursors produces substantial increases in tissue levels of NAD [19], and thus represents an attractive therapeutic strategy, and the outcomes of clinical trials are eagerly awaited [96]. Alternatively, pharmacologically targeting the de novo NAD synthesis pathway in the kidney also seems to be a promising approach in pre-clinical AKI models [52]. However, recent rodent studies suggest that NAD supplementation is not so effective in CKD [26], implying that attention should probably be focused on acute injury.

The aforementioned studies have generated numerous potential targets for intervention that are now being actively explored and have also uncovered key regulators of PT metabolism, such as members of the sirtuin family [14, 81]. Moreover, like NAD supplements, some therapies in development target more than one pathway, which could increase their efficacy. For example, mitochonic acid displays both anti-oxidant and ATP boosting properties [108], while targeting the nuclear pregnane X receptor has pleotropic effects on mitochondrial function [127]. However, the inevitable excitement generated by all these discoveries is slightly dampened by the poor historical track record in the AKI field of successfully translating pre-clinical studies into effective treatments. There are a number of well-recognized generic reasons for this, including issues such as species differences, publication bias, and lack of randomization, blinding, and rigorous statistical analysis [22]. In our view, there are three additional concepts more specific to AKI that have received somewhat less attention to date, but might nevertheless be decisive. These are (1) the importance of heterogeneity in cellular function and metabolism along the PT in determining baseline vulnerability to insults; (2) the diversity of AKI causes and the need to identify insult specific mechanisms to develop precision therapies; and (3) the necessity to integrate alterations in tubular function with changes in whole body homeostasis, in order to devise strategies to reduce associated morbidity and mortality. In the following sections, we will consider each of these issues in turn, and along the way will highlight relevant work performed within the auspices of the NCCR Kidney.ch.

## Heterogeneity of cellular function along the proximal tubule

The nephron is divided into discreet functional segments, and it has long been known that each of these is rendered more or less vulnerable to certain disease causing insults by their intrinsic physiological properties and/or anatomical location [103]. The same concept applies to the sub-sections of the PT. For example, due to its outer medullary environment and hazardous blood supply (predominantly from post-glomerular capillaries), the S3 segment of the straight PT is the major site of damage in ischemia-reperfusion injury [106]. Conversely, experimental application of respiratory chain inhibitors in rat kidneys causes maximal damage in the upstream convoluted segments, because of a lack of anaerobic capacity [9]. Meanwhile, since it endocytoses macromolecules filtered by the glomerulus [104], the early PT (S1) is highly exposed to protein toxins such as myoglobulin [28] and light chains [68].

Nephrotoxins can also enter PT cells directly from the blood via basolateral OATs and OCTs, so axial expression patterns of these transporters are another important factor in determining baseline susceptibility [59, 123]. For instance, OAT1 can transport a wide range of different drugs, some of which are nephrotoxic, such as the anti-viral tenofovir [55]. Antibody staining in mice suggests that OAT1 is highly expressed in the S2 region [7], matching the functional experiments in rabbits mentioned earlier [122]. It remains unclear to what extent this OAT1 expression pattern is replicated in humans [8], and this requires deeper study, but acquiring tissue of sufficient quality to perform quantitative studies is a major practical issue here. In addition to transporters, the abundance of drug metabolizing enzymes (e.g., cytochrome P450s) and anti-oxidant defenses (e.g., glutathione) is also greater in some PT sub-segments than others in rodents and rabbits [91], but detailed human data are again lacking.

The concept of functional diversification along the PT has been further strengthened by live imaging studies, where the dynamic behavior of single cells can be mapped to their anatomical position [76]. For example, we have recently discovered evidence of striking gradients in spontaneous calcium signaling along the mouse PT [75]. Moreover, although S1 and S2 segments are virtually indistinguishable in routine histology sections (unless the former is fortuitously captured leaving a glomerulus), they display very different patterns in metabolic autofluorescence signals [11, 40], many of which are related to mitochondrial metabolism and redox state. In addition, we (A.M.H.) found that inhibition of glucose metabolism had a sizeable effect on cytosolic NAD(P)H levels in S2 segments of mouse cortical PTs in freshly cut kidney slices [11], suggesting that glucose might be important in this region to maintain redox balance via the pentose phosphate pathway, and raising the prospect of heterogeneity in substrate dependence among the segments of the PT.

Older studies performed in vitro with isolated PTs suggested that they *can* utilize a range of different fuels [121], but what they actually *do* metabolize in their native environment is a different matter and will obviously be greatly influenced by substrate availability. For example, recent transcriptomics studies suggest that early (S1) PT cells in mice display a high expression of apical transporters for filtered metabolites such as lactate, pyruvate, and amino acids [13, 98]. Moreover, endocytosis of filtered plasma proteins also represents a potential source of nutrients in this region [34], while albumin bound FAs can hitch a ride into S1 cells this way [80]. Quantitative in vivo studies of metabolite exchange in pigs have also highlighted substantial metabolism of circulating citrate in the PT [46]. Meanwhile, as already mentioned, S2 PT cells have a very high abundance of the *basolateral* metabolite transporter OAT1, which can transport FAs [35], and recent transcriptomic studies in mice also suggest a high expression of the FA transporter CD36 in S2/3 [66]. They are also much more densely packed with peroxisomes [11], which could supply additional lipid fuels via beta oxidation of very long chain FAs. Furthermore, they contain multi-lamellar bodies that probably represent intracellular lipid stores and grossly enlarge in response to sustained high fat feeding [57].

**Fig. 1:**
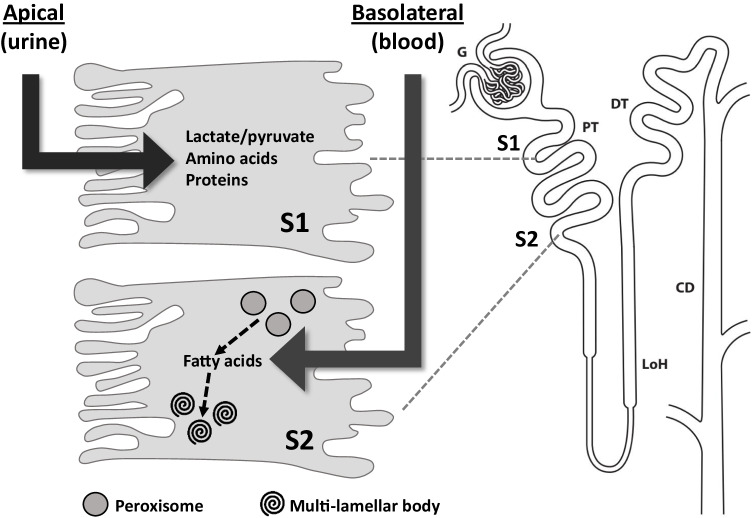
Schematic of metabolic substrate usage along the proximal tubule. The convoluted part of the proximal tubule (PT) comprises of 2 distinct segments (S1 and S2), which display differences in expression levels of membrane transporters for metabolic substrates. Metabolites filtered by the glomerulus are reabsorbed from the primary urine by S1 cells, across the apical membrane. Conversely, cells in S2 have a high abundance of basolateral fatty acid and organic anion transporters, which can import substrates directly from the blood. Moreover, they are more densely packed with peroxisomes that can generate lipid substrates by beta oxidation of long chain fatty acids. Meanwhile, excess free fatty acids within S2 cells can be stored in specialized multi-lamellar bodies found in this region, to prevent potentially harmful lipotoxicity. LoH, loop of Henle; DT, distal tubule; CD, collecting duct

Conceptually, therefore, it can be considered that the early (S1) PT might be to an extent “fed” by the bountiful glomerular filtrate, with luminal substrates such as lactate, proteins (and their cargo), and amino acids taken up by cells from the apical side. Conversely, deprived of this option, downstream segments (S2) have to forage for a living in the bloodstream by directly importing metabolites across the basolateral membrane (Fig. 1). Thus, the oft repeated adage that FAs are the major fuel of the PT is perhaps slightly simplistic and should be revised to take account of axial heterogeneity in metabolite supply, transporter expression, and pathway activity.

The implications of this for understanding the topography of PT damage in AKI need to be carefully considered. For example, in regions with high peroxisomal density, FA oxidation in these organelles could help to compensate for a lack of lipid metabolism in damaged mitochondria, thus limiting harmful rises in free FAs [14]. In addition, there is even evidence that cross-talk between PT segments might occur in sepsis [50], reinforcing the importance of delineating the spatiotemporal evolution of damage in vivo during AKI. How exactly different tubular cells communicate with each other remains far from clear, but release of extracellular vesicles like exosomes is one intriguing possibility, which is currently receiving much attention [88]. The range of usable fuels delivered to the early (S1) PT raises the possibility of a carbon excess under normal conditions that could explain high gluconeogenic activity in this region [124], which is lost in AKI (see below). Finally, the importance of developing a more holistic view of PT metabolism in AKI was nicely illustrated by a recent study showing that effective coordination of mitochondrial biogenesis and mitophagy requires cross-talk with endo-lysosomal system function, and more specifically alignment with regulation of lysosomal biogenesis [70].

## Diversity of insults causing tubular injury

The historical rebranding of “acute tubular necrosis” to AKI was driven by a number of factors, not least the inconvenient fact that necrosis is rarely observed in biopsy specimens [102]. However, one important influence was the success of renaming ischemic heart diseases as “acute coronary syndromes,” which brought numerous benefits for cardiology. These included (1) the recognition that previously distinct clinical entities — like unstable angina and myocardial infarction — can represent different severity points on the same patho-physiological spectrum; (2) the development of effective biomarkers like troponin that changed clinical practice; and (3) the impetus to reorganize services around patient needs and thus improve outcomes. Unfortunately, the adoption of “AKI” has not so far had the same transformative effect in nephrology. Again, there is likely to be more than one reason for this, but a major factor is surely the heterogeneity of insults that can cause AKI in humans [103], in comparison to acute coronary syndromes, which are overwhelmingly driven by atherosclerosis and ischemia.

Clinician scientists working in the AKI field will have no doubt been struck previously by the markedly differing incidences of ischemia-reperfusion injury in pre-clinical studies and patients; being virtually endemic in the former, but mercifully rare in the latter. For sure, renal hypoperfusion can happen during major surgery or hemorrhage, while sepsis probably causes alterations in intrarenal hemodynamics, but these scenarios are not directly comparable with completely clamping the renal artery in rodents. Moreover, although absolute warm ischemia certainly does contribute to tubular damage in the realm of transplantation, a lot of other things happen there too, including prolonged cold ischemia, immune responses, urological problems, and exposure to nephrotoxic drugs, which together vastly complicate the picture. Besides, where warm ischemia does occur in non-transplant settings, evidence suggests that human PTs are more resistant than rodents [90].

Thus, while studies of ischemic AKI have undoubtedly brought some benefits, including reproducibility and the uncovering of generic cellular responses to aerobic insults, transitioning to a more individualized, precision medicine approach necessitates a renewed focus on insults that actually cause AKI in patients and on elucidating *specific* processes critical in each of these [23]. For comparison, a number of very precise molecular mechanisms have now been elucidated in genetic diseases affecting mitochondria in the PT [54, 100], and similar breakthroughs in the AKI field could open the way to more targeted therapies. By analogy, meticulous elucidation of the complement cascade has enabled development of effective antibody treatments for atypical hemolytic uremic syndrome, a rare but important cause of renal failure [39].

### Metabolic changes in specific types of acute kidney injury

**Fig. 2:**
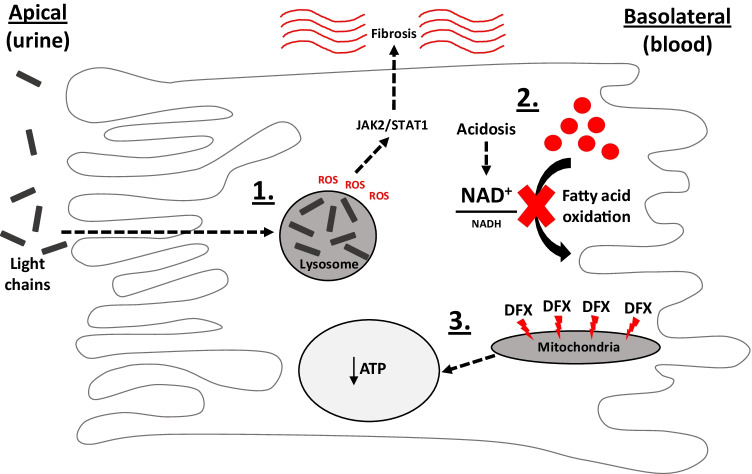
Examples of recently discovered metabolic pathways in acute kidney injury. (1) Uptake of filtered, non-degradable immunoglobulin light chains in proximal tubular cells and accumulation in lysosomes induces reactive oxygen species (ROS) production, which then activates the redox-sensitive JAK2/STAT1 pathway and interstitial inflammation and fibrosis. (2) During acute metabolic acidosis, changes in redox state of the vital metabolic co-factor NADH (towards oxidation) and inhibition of fatty acid oxidation lead to the accumulation of intracellular lipids. (3) The nephrotoxic iron chelator deferasirox (DFX) is highly lipophilic and interacts with the mitochondrial inner membrane, causing severe swelling in these organelles and a decrease in cellular ATP, probably due to partial uncoupling of the respiratory chain

AKI often occurs in the setting of plasma cell dyscrasias like myeloma, due to the toxic effects of free light chain immunoglobulins, which are filtered by glomeruli and endocytosed by the PT. However, the exact reasons why these proteins are harmful to the PT were previously unclear. In a recent breakthrough study, it was shown that lysosomal metabolism of pathogenic light chains induces ROS production, which then activates pro-inflammatory and pro-fibrotic signaling cascades, via the redox-sensitive JAK2/STAT1 pathway [126] (Fig. 2). Critically, depletion of STAT1 in vivo was then protective, demonstrating that elucidation of specific molecular pathways in AKI can facilitate targeted approaches. Moreover, it was recently shown that oxalate excess — which occurs clinically in the setting of ethylene glycol poisoning — results in mitochondrial damage and cell death (necrosis and necroptosis) via induction of the mPTP [82]. Admittedly, this phenomenon occurs mainly in the distal tubule rather than the PT, due to the high luminal concentration and the formation of harmful crystals, but it nevertheless provides another example of a druggable target in a specific form of AKI.

Sepsis is a major cause of AKI in humans but is fiendishly difficult to realistically model in rodents. Therefore, knowledge of the cellular pathogenesis remains limited. Nevertheless, endotoxin administration to mice has been shown to induce mitochondrial swelling in PTs, and an acute suppression of mitochondrial activity, probably driven by downregulation of the master regulator PGC1α [113]. More recently, single cell sequencing has suggested a major transcriptional shift in PTs from solute transport towards immune activation [47]. Meanwhile, evidence of tubular oxidative stress has been reported in a rat peritonitis model, where treatment with a mitochondrial targeted anti-oxidant (MitoTEMPO) was beneficial both for mitochondrial and overall kidney function [3]. Notwithstanding these important observations, we are unfortunately still some way from gaining critical mechanistic insight in human septic AKI. The launching of new large-scale initiatives such as the Kidney Precision Medicine Project — which aim to integrate multiple different types of orthogonal human-derived data — might in time provide the necessary breakthroughs [21], but painstaking experimental work will still be required to dissect out causation from correlation.

### Drug toxicity as a cause of acute tubular injury

The PT secretes a number of xenobiotics from the blood into the urine, and also endocytoses filtered medicines like gentamicin. As such, it is a frequent site of drug induced damage, and nephrotoxicity is thought to account for 25% of cases of AKI [92]. Despite its prominence in the etiology, aside from cisplatin drug toxicity has received relatively little attention in AKI research. Accordingly, in most cases, the underlying mechanisms remain to be properly elucidated at a cellular level, although mitochondria in the PT have long been suspected as major targets [44]. With improvements in pre-clinical models and cellular analysis techniques, this is beginning to change [18].

Using live cell imaging, we (A.M.H.) recently identified a mitochondrial mechanism of toxicity from the oral iron chelator deferasirox [33], which is associated with PT defects in humans [24]. Since other medically used iron chelators are not apparently nephrotoxic, this raised the clinical suspicion that deferasirox might have an off-target effect, and our findings suggested that this is indeed the case. We observed that deferasirox — but not other chelators — induces acute swelling of mitochondria and ATP depletion within PT cells, probably due to an interaction between the drug and the inner mitochondrial membrane, leading to a partial uncoupling of the respiratory chain [33] (Fig. 2). Of note, the chemical properties of deferasirox (a lipophilic weak acid) that are likely responsible for this phenomenon are shared by some other nephrotoxins (e.g., non-steroidal anti-inflammatories), raising the possibility of a common mechanism [84]. In contrast, some other well-established nephrotoxins exert quite different effects on mitochondria. For example, the anti-viral tenofovir induces mitochondrial hypertrophy, rather than swelling, and marked changes in cristae morphology [41], hinting at a quite distinct pathological process. Importantly, elucidating toxicity mechanisms can reveal new paradigms for prevention. For example, we discovered that the harmful effects of deferasirox on mitochondria are mediated by the free form of the drug, and that binding to iron or albumin can substantially ameliorate toxicity, at least in cells [33]. Extrapolating from this to humans, it is possible that iron store levels and blood protein concentration might determine baseline susceptibility to toxicity in humans, but this remains to be proven.

The preceding examples underscore the principle that mechanisms of mitochondrial injury in the PT are likely to be insult specific. While the downstream consequences might converge on common pathways (like mitophagy, say), it seems evident that developing effective treatment strategies will be contingent on a more precise understanding of the initial upstream events. By analogy, reducing intra-glomerular pressure with renin-angiotensin-system blockers is broadly beneficial in many different glomerulopathies, regardless of the exact cause, but this has not precluded an intensive search for disease specific molecular changes that might be more precisely targeted in tandem.

### Tubular targeted therapies

The PT is armed with an array of cell membrane transport mechanisms that can potentially be harnessed, either to introduce novel therapies or to block the uptake of toxins. For example, PT cells avidly endocytose macromolecules filtered from the glomerulus (via the megalin/cubilin system), and a recent study showed that this provides a conduit to deliver anti-oxidant nanoparticles to the PT, which provide protection in a model of septic AKI [115]. Conversely, inhibition of megalin-mediated myoglobin uptake prevents tubular damage in rhabdomyolysis [77]. PT cells in vivo have a close structural and functional relationship with surrounding stromal cells [42], and tubulo-interstitial cross-talk might also be exploited to enhance metabolic function in damaged PTs. Along this line, injection of human mesenchymal stromal cells into mice seems to stimulate mitochondrial biogenesis in the PT and improves mitochondrial function in a mouse model of cisplatin AKI, possibly via the activity of sirtuin 3 [93].

### Precision diagnosis with biomarkers

Accurate diagnosis and targeting of different types of AKI in humans will be massively enhanced by the development of specific blood or urinary biomarkers, and this has been an area of intense research interest in the last few years. Some urine biomarkers arise predominantly from defined tubular regions (e.g., kidney injury molecule 1 from the PT, uromodulin from the thick ascending limb of Henle) and can thus indicate regions of damage along the nephron [120]. Moreover, older studies suggested that alkaline phosphatase might denote damage in S3 in humans [87]. However, we still remain some way from being able to use biomarkers to localize injury to sub-segments of the PT or to denote activation of insult specific cell damage pathways. Nevertheless, they do provide the opportunity to detect tubular injury well before rises in blood creatinine, and thus to predict AKI progression in settings such as cardiac surgery [120].

## Integration of acute kidney injury with whole body homeostasis

More than any other organ, the kidney maintains the milieu intérieur, upon which complex organisms are utterly dependent, by regulating processes such as osmolarity, bone and mineral homeostasis, hemoglobin concentration, and acid-base status. It therefore follows that acute loss of renal function has widespread consequences for patients, which in turn might explain the prominence of AKI in critical illness. However, the exact homeostatic disturbances that drive poor patient outcomes in AKI have remained opaque, which generates substantial equipoise in patient management. For example, arguments are still raging as to whether early correction of dyselectrolytemia with renal replacement therapy is beneficial or not [48].

### Glucose homeostasis in acute kidney injury

**Fig. 3:**
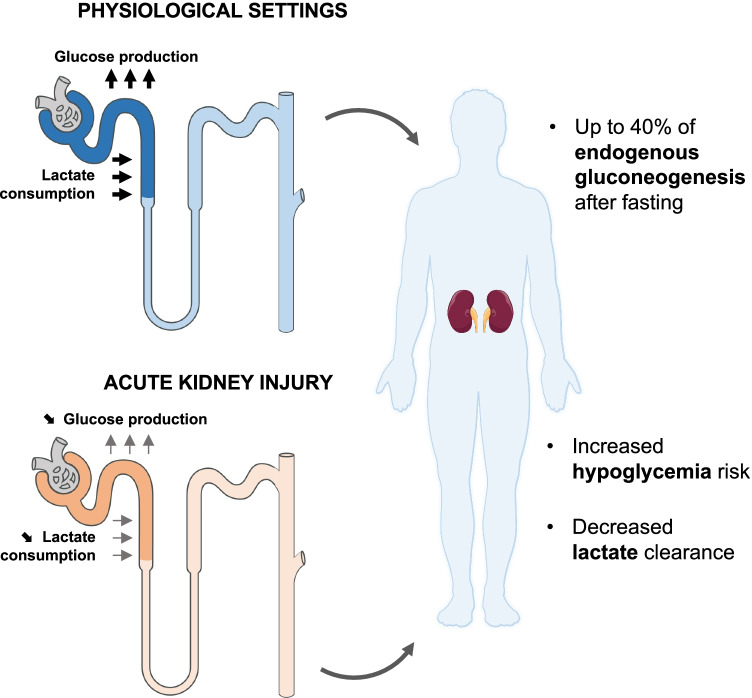
Alterations in systemic glucose homeostasis in acute kidney injury. Under normal physiological settings, the kidney contributes up to 40% of body gluconeogenesis after fasting, by converting lactate to glucose in the proximal tubule. During acute kidney injury, this process is dramatically downregulated, leading to decreased lactate clearance and increased risk of hypoglycemia

Glucose is a vital fuel for various organs, including muscle and brain, and the kidney contributes up to 40% of systemic gluconeogenesis in the fasted healthy state, mainly via the metabolism of lactate in the PT [29]. In a recent study, one of the authors (S.d.S.) demonstrated that this essential metabolic function of the kidney is dramatically altered during AKI [61]. In both experimental models of ischemia-reperfusion and biopsies from kidney allograft patients during the reperfusion phase, striking downregulation of the whole gluconeogenetic pathway was observed. This was coherent with a reduced ability to clear lactate and produce glucose in the experiment setting, and with lower glucose and higher lactate serum levels in patients with AKI. Crucially, these metabolic alterations were themselves associated with increased mortality risk. Thus, AKI not only alters lipid metabolism but also has a profound effect on glucose homeostasis (Fig. 3), which provides at least one plausible link between tubular cell fitness and patient survival.

The importance of the kidney in glucose metabolism has also been highlighted by the widespread usage of sodium-glucose transporter 2 (SGLT2) inhibitors. While protective against progression of CKD, there have been concerns that these drugs might cause AKI in some patients, possibly due to natriuresis and volume depletion [36]. However, more recent meta-analysis of multiple studies actually suggests a protective effect [79], perhaps due to redistribution of the solute transport load along vulnerable tubules. Experiments in mice have also suggested direct benefits of SGLT2 inhibitors on PT cell metabolism in diabetes, including preventing suppression of sirtuin 3 and aberrant glycolysis [63], and possibly also promotion of ketone body metabolism [112]. Of note — and relevant to the earlier discussions on tubular physiology — the sodium-glucose transporters SGLT1 and 2 display striking axial patterns in expression in the PT [116]. Moreover, evidence suggests that SGLT2 expression and activity is downregulated in rodent PTs in response to ischemia-reperfusion injury [116], and loss of tubular glucose reabsorption might further contribute to disturbances of systemic blood glucose that occur in human AKI.

### Organic solute excretion in acute kidney injury

As mentioned previously, a major function of the PT is to secrete organic solutes into the urine that are poorly filtered by the glomerulus (e.g., due to albumin binding). A number of organic anions — including indoxyl sulfate, indoxyl acetate, kynurenine, kynurenic acid, and p-cresyl sulfate — are putative uremic toxins, which are generated by the gut microbiome and normally removed from the blood by basolateral OATs expressed in the PT [117]. Loss of renal function therefore causes a build-up of these substances in the blood, and this might contribute to the high burden of cardiovascular disease in patients with CKD [99].

Since a number of substances transported by the OAT system are potential fuels (including FAs) [35], and mitochondria within the PT can both metabolize and generate uremic toxins [95], this represents another important intersection between tubular metabolism and whole-body homeostasis. The clinical relevance of this was highlighted by a recent study demonstrating association of impaired tubular organic solute secretion with worsening of a combined renal end point (including death) in patients with critical illness [6]. Moreover, tubular secretion is also a vital elimination pathway for many drugs (e.g., antibiotics and antivirals) that are widely used on intensive care units. Improving understanding of how this system is altered in AKI could lead to more accurate drug dosing, thus reducing iatrogenic harm and further improving patient outcomes.

### Acidosis as a driver of tubular injury

Loss of renal function undoubtedly disturbs body homeostasis, but the high prevalence of AKI in intensive care units also raises the possibility that the reverse may be true, i.e., that some perturbations in normal blood consistency might themselves be harmful for the kidney. As an example of this concept, a recent clinical study suggested that correcting metabolic acidosis in patients with critical illness substantially improves renal outcomes [45], which is line with numerous studies suggesting a benefit in CKD [83], and suggests that acidosis is somehow injurious to the kidney.

To explore the cellular events underlying this intriguing observation, the group of one of the authors (A.M.H.) recently performed live cell imaging studies in mouse kidney cortex and found that acidosis induces acute metabolic changes in PTs, including in mitochondrial NAD redox state, respiratory chain function, and intracellular lipids [12] (Fig. 2). Importantly, these phenomena were associated with tubular damage characteristic of AKI, and functional defects in filtered solute transport could be substantially ameliorated by bicarbonate therapy or NAD supplementation. Thus, this provides yet another example that integrating clinical knowledge (i.e., what actually triggers AKI in patients) with mechanistic insight from basic research studies can ultimately generate new treatment strategies that are viable in humans.

## Conclusions and future directions

AKI is most frequently caused by damage to the PT and is associated with substantial morbidity and mortality. Much has been learnt about the metabolic changes that take place in PT cells, and numerous potential therapeutic targets have been identified. However, despite a long history of promising pre-clinical studies in AKI, something always seems to get lost in translation, and treatment options for patients have remained limited (to put it mildly). There are a number of possible reasons for this, some of which are rather generic and equally germane to other organ systems and diseases, not least the often underappreciated conceptual differences between exploratory and confirmatory research [1]. Nevertheless, in this article, we have highlighted three specific areas that we believe deserve renewed consideration in the AKI field.

First, delineating more precisely the heterogeneity of cellular function along the PT will enhance understanding of baseline vulnerability — and prediction of response — to metabolic insults in the different segments and will probably also shed new light on the nature of adaptive remodeling post injury. Importantly, it remains to be established to what extent the striking functional topography of the PT in experimental rodents is replicated in humans. Second, elucidating exact cellular mechanisms in specific types of AKI could facilitate more targeted therapeutic approaches to limit damage, which might even be combined with broader interventions like manipulating mitophagy or mitochondrial biogenesis to speed recovery. Lastly, metabolic changes occurring within tubules during AKI should be integrated with alterations in whole-body homeostasis (e.g., regulation of glucose, organic solutes, and acid-base status) to improve patient outcomes, a concept nicely embodied by the full title of the NCCR network: “Kidney Control of Homeostasis.”

Moving forward, a more personalized era could be envisaged, whereby patients with AKI would undergo detailed assessment to establish the 6 “W”s of their tubular injury: Which insult is predominantly causing the injury? Where along the tubule is the damage? When did it start? Why is it occurring in this specific patient? What are the systemic consequences? Will the tubules adapt and/or recover? Realization of this ambitious aim will obviously require rather more sophisticated measurements than blood creatinine and urine output. Some of the knowledge gap could be filled by existing methodologies like functional MRI [105] and urinary biomarkers [78], but, realistically, newer technologies will be required to obtain the requisite game-changing insights [86]. The design and development of these will be guided by deepening pre-clinical understanding of the precise cell and molecular events that occur in different types of AKI. This in turn will most likely necessitate multi-disciplinary working with specialists currently outside the kidney field, the generation of more sophisticated in vitro models, and the integration of various different data sets (gene expression, metabolomics/proteomics, intravital imaging, etc.) [20]. Meanwhile, an increased usage of larger animals in the research pipeline might help to bridge the species gap between rodents and humans [89]. Finally, because more than one metabolic pathway can be perturbed in AKI, multi-target drugs might ultimately be more effective than single silver bullets [43].

Analogies are often drawn between difficult-to-crack diseases and military conflicts (the “war on cancer,” etc.). Historical study of the latter suggests that to break any longstanding stalemate new fronts have to be opened up. Understanding how metabolism changes along PTs in an insult specific manner — and the consequences of this for the topography of damage and remodeling, the genesis of biomarkers, and whole-body homeostasis — represents one promising line of inquiry in AKI research.
